# Archaeozoological Analysis of Bovine Proximal Phalanges from Medieval and Early Modern Wrocław

**DOI:** 10.3390/ani16142234

**Published:** 2026-07-18

**Authors:** Aleksander Chrószcz, Dominik Poradowski, Joanna Klećkowska-Nawrot, Joanna Wolińska, Kamilla Pawłowska, Krzysztof O. Stegmann, Dominika Kubiak-Nowak, Vedat Onar, Zahide Sena Güneş Kaya

**Affiliations:** 1 Department of Biostructure and Animal Physiology, Faculty of Veterinary Medicine, Wrocław University of Environmental and Life Sciences, ul. Kożuchowska 1, 51-631 Wrocław, Poland; 2 Doctoral School, Wrocław University of Environmental and Life Sciences, ul. C.K. Norwida 25, 50-375 Wrocław, Poland; 3 Department of Palaeoenvironmental Research, Institute of Geology, Adam Mickiewicz University Poznań, ul. B. Krygowskiego 12, 61-680 Poznań, Poland; 4 Regionalny Zarząd Gospodarki Wwodnej, ul. C.K. Norwida 34, 50-950 Wrocław, Poland; 5 Department and Clinic of Surgery, Faculty of Veterinary Medicine, Wrocław University of Environmental and Life Sciences, pl. Grunwaldzki 51, 50-366 Wrocław, Poland; 6 Department of Anatomy, Milas Faculty of Veterinary Medicine, Muğla Sıtkı Koçman University, Beçin Mah., Üniversite Cad. No: 248200, Milas, Muğla 48000, Türkiye; 7 Department of Architecture, Faculty of Architecture, Istanbul University, Beyazıt, Istanbul 34452, Türkiye

**Keywords:** cattle, long pastern bone, archaeozoology, osteometry, metal alloys, computed tomography

## Abstract

This study focused on an archaeozoological analysis of the morphology and osteometry of bovine proximal phalanges. The examined material consisted of 446 complete specimens recovered from an archeological site in Wrocław, Poland (St. Vitus and St. Catherine Streets and New Market Square). The assemblage was divided into eight chronological groups spanning the period from the Early Middle Ages to the Modern Era. The archaeozoological investigation included osteometric measurements and an assessment of morphological variation. Observed changes in bone dimensions were interpreted in relation to patterns of animal exploitation, successive stages in the development of proto-urban and urban settlement in Wrocław, and environmental influences. A critical evaluation of the osteometric data demonstrated that the greatest length of the proximal phalanx should not be used as an additional proxy for estimating withers height when complete metapodial bones are absent from an archeological assemblage. Furthermore, computed tomography (CT) analysis revealed the presence of metal alloys within the medullary cavity of several bones, indicating intentional human modification of the material in the past.

## Simple Summary

The fingers of cattle consist of three bones (excluding three small sesamoid bones); the longest, the long pastern bone, was chosen for analysis. The study consists of a comparison of the shape and dimensions of the bones, which were influenced by how animals used them. The material (446 samples) came from an archeological site in Wrocław, Silesia, Poland and was dated to between the Early Medieval and Modern periods. The aim of the study was to describe cattle exploitation in the above-mentioned historical period. Moreover, the potential use of an animal height estimation method based on the measurements of the bones was evaluated. The results showed that the influence of animal use on the animal skeleton did not cause severe changes to the bones. The height at withers computed using the above-mentioned method cannot be successfully used with non-modern material. The computed tomography proved that some samples caused interferences to be visible in scans, which was the result of metal alloys existing within the bones.

## 1. Introduction

Archaeozoological studies of the bovine bone assemblages from the archeological sites located in Poland showed that Medieval and Early Modern cattle were shorter than modern animals, not exceeding a height at withers of 100–120 cm [1,2,3,4,5,6,7,8,9,10,11]. Since the beginning of proto-urban Wrocław complex development, cattle constituted an unquestionably important structural element of livestock and draught animals. Moreover, the population of this species responded to a constantly growing demand for milk, meat, animal production waste (horns and hoofs), and hides due to an increase in human population density, level of urbanization, and political reality. The historical Duchy of Silesia had three neighbouring monarchies that competed with each other: Bohemia, Poland and Germany. This meant there were intersecting trade routes and, therefore, the economic potential of Wrocław (the capital city of Silesia) significantly influenced the history of the town and its population [3,5,12,13,14,15,16,17,18]. Along with the chartering process of the medieval town based on Magdeburg Rights, subsequent urban organization and socio-economic development, as well as the gradual diversification of pig and cattle meat products, can be observed [3,4,5,12,19,20,21].

**Figure 1:**
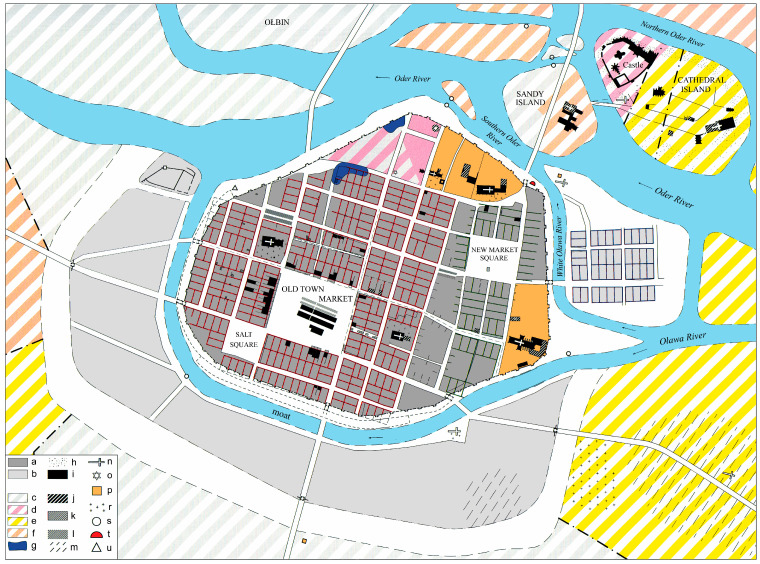
Chartered medieval Wrocław and proto-urban settlements (Cathedral and Sandy Islands) around 1300 AD [22]. a—urban area; b—non-urban area incorporated into the town in the second half of the 13th c.; c—municipal property; d—ducal property; e—ecclesiastical property; f—monastic property; g—confirmed Jewish quarter; h—early earth-and-timber rampart; i—confirmed masonry structures; j—reconstructed masonry structures; k and l—attested timber buildings; m—settlement; n—approximate location of a church; o—synagogue; p—hospital; r—cemetery; s—mill; t—inn; u—slaughterhouse.

According to a long-standing legend, the first settlement was named after Vratislaus I, Duke of Bohemia (915–921 AD), of the Czech Přemyslid dynasty, although Bohemia probably did not control Wrocław until about 945 AD. The first written mention of Wrocław appears in the Thietmar Chronicle, which records the promotion of an episcopal see by the Roman Emperor Otto III and Boleslaus I the Brave, future King of Poland, during the Congress of Gniezno (1000 AD). Wrocław was probably first chartered under Magdeburg law, probably during the reign of Henry I the Bearded, House of Piast, Duke of Silesia (ca. 1226 AD). The chartered settlement may have been near present-day New Market square (Ger. Neumarkt), although Old Town Market square (Ger. Ring) is considered more likely; the absence of the charter document leaves the issue unresolved. The second urban charter was granted in 1242 AD, right after the Mongol invasion of 1241 (Duke Boleslaus II the Bold). Finally, in 1261 AD, under Duke Henry III the White, the charter model was introduced, apart from the above-mentioned spots, in the New Market square quarter, transforming the older proto-urban craft and market settlement known as *ad sanctum Adalbertum* (Figure 1). The New Town (Ger. Neustadt) was chartered in 1261–1263 AD, and all three urban centres were united in 1327 AD [3,5,12,13,14,15,16,17,18].

In medieval Poland, houses were not merely residential buildings but multifunctional spaces in which various economic activities were carried out. Agriculture and animal husbandry were fundamental components of daily life in both rural and urban areas. Consequently, areas associated with storage, production, and livestock-related activities were commonly integrated into residential complexes. However, the available archeological evidence is not always detailed enough to demonstrate that animal shelters followed a uniform spatial layout or architectural plan. Most likely, land used for agriculture and animal husbandry was gradually shifted to the suburbs (outside the city walls), as a consequence of late medieval urban organization [15,16,17,18]. Studies of the spatial development of Polish towns indicate that medieval urban centres maintained strong connections with the rural economy for a considerable period. Despite ongoing urbanization, economic activities in many towns extended beyond trade and craft production to include agricultural practices and animal husbandry [23].

Although the Renaissance and early Modernity produced important works on domesticated animals used in agriculture, their authors were generally not veterinarians but mainly people of different national provenience who had varying degrees of theoretical and practical knowledge of animal use, care, and basic treatment [24,25]. During the 18th and 19th centuries, this body of knowledge was gradually evaluated and incorporated into modern veterinary sciences by adepts trained in Lyon from 1764 AD onward [26].

The quarter bounded by St. Vitus, St. Catherine streets and New Market square was continuously occupied as part of the settlement. Changes in the proximal phalanges osteometry may therefore reflect the transition from a proto-urban settlement to a chartered Medieval town and, subsequently, towards Modernity. Simultaneously, the changes in the inhabitants’ economic status, the political situation in Silesia, and ethnic and religious transformations may also have influenced cattle breeding, use and trade and may be reflected in the archaeozoological records. The study aimed to analyze the morphology and morphometric characteristics of the proximal phalanges recovered during an archaeozoological investigation of the bone material from Wrocław’s Old Town. In addition, height at withers computed using the method of Chrzanowska and Wagner [27] method was evaluated against the archaeozoological bone assemblage.

## 2. Material and Methods

**Figure 2:**
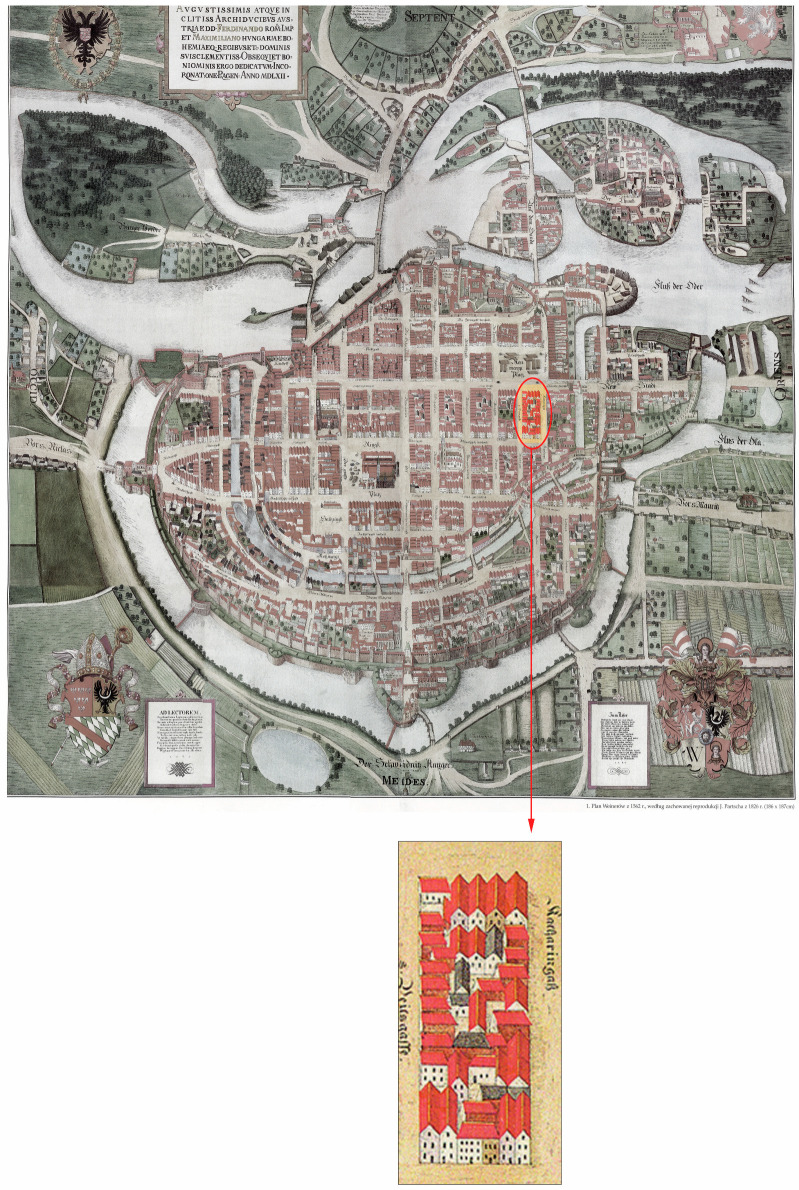
Wrocław on Barthel Weiner’s 1562 AD plan, reproduced by Christian Friedrich Partsch in 1826 AD, after Wrocław on plans from 16th–20th century, Wrocław 1999. The archeological plot was located between the New Market square and St. Catherine and St. Vitus streets (enlarged below).

The accessible bone remains were unearthed from an archeological site located in quarter bounded by: St. Vitus Street, St. Catherine Street (Ger. Ziegengasse, Katharinenstrasse), and New Market square (Ger. Neumarkt) (Figure 2). The material came from stratigraphic units dated back from the Early Middle Ages to the Modern period. Anatomical terminology followed *Nomina Anatomica Veterinaria* [28].

The animal bone assemblage comprised 446 artefacts, including 9 bones poured with metal alloy, and was identified in the visual-comparative analysis using the reference collection of animal skeletons held by the Division of Animal Anatomy, Wrocław University of Environmental and Life Sciences. The proximal phalanges of cattle (*phalanges proximales bovis*) were selected for further analyses. To asses chronological differences in the measured variables, the accessible material was divided into eight chronological groups (Table 1).

Osteometric examination was used to develop a simplified description of the historical cattle morphology. The greatest length of the proximal phalanx (GLpe) was used to calculate the height at withers (HW-p). Although the method of Chrzanowska and Wagner [27] for estimating HW-p was developed from modern cattle skeletal material, it was applied to the accessible archaeozoological bone remains in this study. The resulting estimates were compared with the height at withers (HW-m) calculated from metapodials [30] to evaluate the effectiveness of the first method in archaeozoological analyses.

Radiological imaging diagnostics, in the form of computed tomography (CT), was additionally used to confirm the presence of poured metal alloys within the medullary cavities of bones. All scans were acquired with a 64-slice, 128-layer Siemens go. TOP CT scanner (Siemens AG, Munich, Germany) along the longitudinal axes of the bones in the cranial and caudal directions. The exposure parameters were set to 120 kV and 60 mAs. Image cross-sections were reconstructed using a bone filter at a slice thickness of 0.6 mm. The CT images were post-processed using multiplanar reconstructions in the sagittal, dorsal, and transverse sections and the three-dimensional image functions provided by Siemens’ syngo.via VB80A software. In this study, CT scanning was used specifically to detect the metal alloys in the bone material. Because the assemblage included 446 specimens and the complete description of the bone architecture would exceed the scope of this manuscript, those radiographic findings will be reported separately in a paleopathological study.

The data were analyzed using Student’s *t*-test in StatisticaPL 10.0 software (StatSoft, Kraków, Poland). For each measured dimension, the arithmetic mean and standard deviation were calculated to summarize central tendency and dispersion. Maximum and minimum values and variance were also determined.

The results were documented and are presented in tables and figures. Macroscopic photographs were acquired with a digital camera (Sony Alpha DSLR-A300, zoom objective 18–70 mm, Sony Corporation, Tokyo, Japan) and subsequently processed using dedicated software.

## 3. Results

### 3.1. The Osteometric Examination and the Height at Withers Calculation

**Figure 3:**
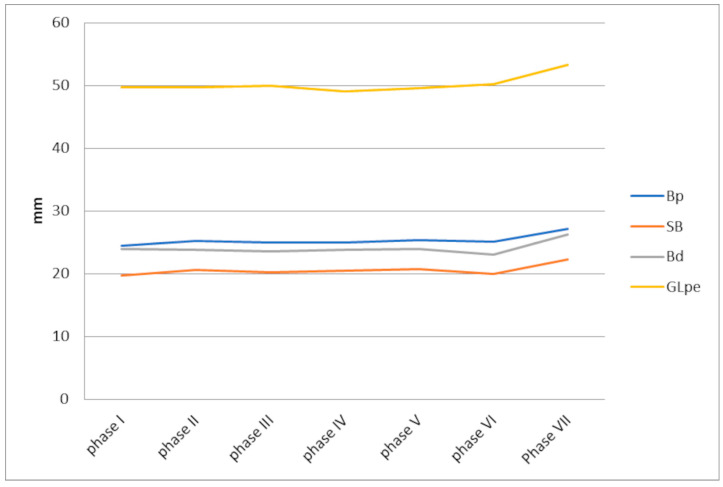
The osteometric measurements [mm] across successive phases of chronology. GLpe—greatest length (peripheral), Bp—breadth of the proximal extremity, SB—smallest breadth of the diaphysis, and Bd—breadth of the distal extremity.

The mean values and standard deviations for the subsequent osteometric parameters are presented in Table 2, and changes across chronological phases are shown in Figure 3. The greatest length of the proximal phalanx was also used to calculate the height at withers according to Chrzanowska and Wagner [27]. The remaining variables (Bp, SB and Bd), together with GLpe, are routinely used in archaeozoological studies of bone morphology following the methodology introduced by von den Driesch [29].

**Figure 4:**
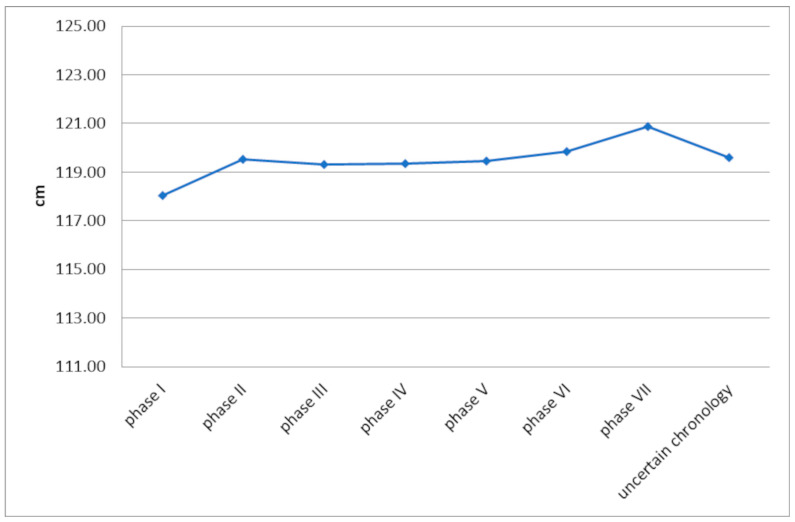
Changes in estimated height at withers (HW-p) across successive chronological phases [cm]. Whiskers indicate the standard deviation. Proximal phalanx dimensions were moderately uniform across phases; all measurements increased in the seventh phase (Figure 3). HW-p, calculated from GLpe (Figure 4) showed the greatest variation in the first phase, remained stable in the third–fifth phase, and increased in the sixth and seventh phase. The method was developed using the modern cattle bone assemblage sourced from the individuals with known height at withers. Chrzanowska and Wagner [27] indicated that it is applicable to the GLpe range values typical for modern cattle and characterized it as a supplementary method that tends to overestimative the height at withers relative to Całkin’s commonly used in the archaeozoological practice HW-m method [31]. HW-p values were compared with HW-m values [30]. GLpe-based estimates did not correspond to HW-m values obtained using Całkin’s reference metapodial method (Figure 5) [31]. Therefore, HW-p cannot serve as an alternative method for the height at withers estimation in historical cattle because archaeozoological GLpe values fall below the range used to develop the formula. This limitation also applies to assemblages in which complete metapodia are unavailable for morphometric studies.

Comparison across chronological phases showed that Bp, SB and Bd remained relatively stable over time. GLpe increased slightly in the fifth and sixth phase and more markedly in the seventh phase (Figure 3). The similarity of these values indicated that the proximal phalanx morphology remained uniform and did not show severe pathologies in the locomotor system typical of overloading or intensive draught animal use. The increase in GLpe during the final chronological phase may reflect greater animal height at withers. Therefore, following the method introduced by Chrzanowska and Wagner [27], GLpe was used to estimate approximate HW-p. This variable, together with its standard deviation, is presented in Table 3 and Figure 4.

### 3.2. The Computed Tomography as a Tool of Metal Alloys Identification

CT scanning is routinely used to examine internal bone structure both in veterinary diagnostics and archaeozoology. In this study CT images were reconstructed from a series of two-dimensional radiographs acquired as an X-ray tube rotated around each specimen [32,33]. The analysis was limited to evidence of intentional human modification within the bone assemblage. The metal alloy deposits within the medullary cavities of bones produced clearly visible imaging artefacts as an image interference (Figure 6 and Figure 7).

## 4. Discussion

The study was limited to the proximal phalanges from the *acropodium* of domestic cattle. In limb anatomy, the *acropodium* (the skeleton of hand and foot) comprises three digital segments (phalanges) and the sesamoid bones. The proximal phalanx (*os compedale*, long pastern bone) and the middle phalanx (*os coronale*, short pastern bone) are long bones with a bone shaft (*diaphysis*), two extremities (*extremitates*), and a medullary cavity (*cavum medullare*). The distal digital phalanx (*os ungulare*) is classified as an irregular bone (*os multangulum*), and it is covered by the hoof (*ungula*) in vivo [34,35]. This study examined bovine proximal phalanges archeozoologically, including osteometric analysis, height at withers estimation, and evidence of intentional bone modification and use, to contribute to the reconstruction of animal–human-environment relationship in the past.

### 4.1. Osteometric Examination—Measurements of the Proximal Phalanges

Previous studies have frequently examined the morphology and morphometry of bovine phalanges. Dottrens [36] analyzed differences in osteometric variables according to the anatomical position of the phalanges (the thoracic versus pelvic limb and the third versus fourth digit). A critical archaeozoological study by Bartosiewicz [37] substantially limited the reliability of assessing anatomical position using the evaluated criteria. Both proximal and middle phalanges are the long bones characterized by early ossification of the epiphyseal cartilage (approximately 18 months) [34,35,38]. Although the proximal phalanges of pelvic limb are usually longer and more slender than those of the thoracic limb, position of the phalanx (medial or lateral) also substantially influences its proportions [36,37]. The animal’s age, body weight, sex, and animal labour should also be considered when assessing the anatomical position of each bone [37]. The accessible bone material was unearthed during the archeological excavation and classified as consumptive waste. The phalanges could not be attributed to specific individuals or assigned reliably to the thoracic or pelvic limbs. Therefore, the bone assemblage was analyzed as a whole.

Standardized methods are required to compare morphological trends in cattle across centuries or geographic regions. These requirements also apply to methods used to estimate the height at withers [39,40]. Published studies show that animal labour can produce skeletal changes with varying severity [1,2,3,4,5,41,42]. The osteometric analysis indicated that the bones from the Wrocław site represented a broad large group of animals, but the morphology, proportions, and dimensions of the bovine long pastern bones were relatively uniform. The highest standard deviation (4.21) occurred for GLpe in the final chronological phase (Table 2).

Across chronological phases (Figure 3), GLpe decreased slightly in the fourth phase of chronology and then increased gradually in the next two phases (fifth and sixth). The fourth phase of town development (1261 AD–half 14th c.) was a pivotal period in the history of New Market area because it included the foundation of chartered town, the organization of burgher’s plots and transition from proto-urban to urban left-bank Wrocław. The pattern suggests that the late medieval and early modern city (half 14th c.–17th c.) not only stabilized the social and economic life of its burghers but may also have permitted greater phenotypic expression of the animals’ genetic potential or reflected the introduction of new breeds. The pattern could also reflect an increasing proportion of oxen in the livestock [3,4,39]; however, archaeozoological analysis of the bovine skeletal assemblage indicated a predominance of bulls [30].

Bp and Bd values were similar across successive phases (Figure 3). The lowest Bp values probably came from young animals, whereas the highest values may be typical of large bulls and oxen, particularly for bones from the thoracic limbs, which support most of the animals’ body weight [39,43,44]. The base of the proximal phalanx (the proximal extremity) had a typical rectangular-trapezoidal shape [34,35]. Flattening of the extremity and its articular surfaces may indicate uneven body weight distribution between the limbs caused by long-term animal use, orthopedic disease, or degenerative processes [43,44]. The morphology of the examined bovine proximal phalanges did not show bone changes typically interpreted as severe pathology or animal overloading associated with intensive use as draught animals in archaeozoological and paleopathological studies [41,42,45,46,47]. These findings suggest that most phalanges came from healthy animals selected from local cattle populations or imported and slaughtered to meet urban demand for beef. A broader study of Wrocław’s animal economy indicated rational herd management, with animals that did not meet herders’ expectations removed from secondary-product production. Trade routes intersecting near the town also facilitated intensive importation of cattle, as documented in historical sources, and reflected urban economic importance [1,2,3,4,5,18,30,48].

For SB, the osteometric landmarks are clearly defined in young, healthy animals without any diseases of the locomotor system, and the measurements can be obtained readily. In older animals, this part of the diaphysis may thicken because of bone remodelling at insertion points of the digital tendons and ligaments [34,44]. Similar SB values across all chronological phases may reflect the continued importation of young cattle for meat production, possibly for wealthier consumers. The economic status of the burghers and the demand for high-quality meat may have enabled optimized slaughter practices and reduced reliance on older, fully exploited cattle for consumption. An alternative explanation concerns the beef trade and the locations of butcher stalls. The findings may suggest that stalls near New Market square supplied higher-quality meat, whereas older animals were slaughtered elsewhere in or near the city for meat and hide production and sell. Bone changes and bone tissue remodelling near tendon and ligament insertions are frequently observed in the archaeozoological assemblages in which heavy animal labour has been documented [41,42,45,46,47]. The absence of marked differences in Bp, SB and Bd is consistent with the absence of severe lesions of the metacarpophalangeal/metatarsophalangeal joints, the proximal interphalangeal joint, and the proximal phalanx diaphysis [44].

Variation in GLpe may be explained by the anatomical position of the proximal phalanx within the skeleton of hand or foot, in which the axis of hand and foot (*axis manus et pedis*) lies between the third and fourth digit [34,35]. Uneven distribution of the body weight between the thoracic and pelvic limbs, together with the distinct biomechanical roles of the third and fourth digit, generates physical forces that act on living bone tissue. These factors affect postnatal development, ossification of the epiphyseal cartilage, and remodelling of bone architecture of a living organ. Because they strongly influence phalangeal morphology, precise assignment of isolated phalanges to the thoracic or pelvic limb is difficult [36,37]. Anatomical and radiographic studies of the hand and foot skeleton in modern cattle have shown that the fourth digit is longer in both the thoracic and pelvic limbs. Biomechanical analyses have also demonstrated the dominant role of the fourth digit in the pelvic limb and approximately equal roles of the third and fourth digit in the thoracic limb in supporting animal body weight [43]. Increased loading of the fourth digit in the foot has been identified as a major predisposing factor for sole ulcers (Rusterholz ulcers) in cattle [43,44,49]. Taken together, the findings suggest that the cattle population of medieval and early modern Wrocław had relatively good limb health, without severe lesions evident in the bone architecture.

A comprehensive description of the role of domestic animals in Wrocław’s proto-urban and urban settlements is beyond the scope of this study. The accessible bone assemblage comprises more than 66,000 artefacts and was dominated by domestic species and cattle skeletal remains; swine bone fragments predominated in the fifth phase and occurred in similar proportions in the sixth phase [30]. The distribution of identified specimens indicates that beef was the most important source of meat in Wrocław’s history. Combined archeological analyses of settlement development and transformation make it possible to address whether livestock were raised within the city walls. During the first and second chronological phase, the pre-urban early medieval settlement and the *ad Sanctum Adalbertum* craft and market settlement, animal breeding and herding were practiced near humans dwellings. Later, in third–fifth phase, intensive chartering shifted livestock areas to suburbs. Construction of the city walls, human population growth, and the formation of burgers plots substantially reduced the urban space available for domestic animals. The late medieval and early modern periods (the fifth–seventh phase) were characterized by the urban transformations typical of large cities [15,16,17,18]. The medieval municipal authorities prohibited not only livestock herding within the city walls but also the driving of herds through the city [26]. These observations support the hypothesis that farm animals were gradually removed from the urban space and relocated to developing suburban areas. The unchartered right-bank settlement of Ołbin had hosted a cattle market since the Early Middle Ages [15,16,17,18]. Livestock imports, facilitated by trade routes near the city and therefore played a crucial role in the urban meat supply. The sex ratio showed a predominance of bulls in the first to fourth and seventh phases, and age of slaughter was generally 24–42 months, except in the sixth phase [30]. Together with the archeological context, and consumptive character of the bone assemblage, and the presence of animal-bone workshops through the third phase, these findings indicate that cattle exploitation in Wrocław was efficient and rational. The system was probably disrupted only temporarily during wars and crises, reflecting the high economic resilience of the Silesian capital.

### 4.2. Analysis of Height at Withers in Cattle from 11th to 19th Century

Godynicki [50] and Godynicki and Godawa [51] performed statistical analyses of the metapodia and phalanges of modern roe deer and red deer. Like Chrzanowska and Wagner [27] in their study “*Statistical analysis of the morphological diversity of the proximal phalanx of cattle*”, they identified a strong correlation between the greatest length of the metapodium and GLpe. These relationships were used to develop regression equations for indirect estimation of living animal height at withers with a high degree of probability. Chrzanowska and Wagner [27] evaluated the correlation and regression relationship between GLpe and HW-p in cattle. Their material consisted of bones from modern cattle separated by digit (the third and fourth digit) and limb (thoracic or pelvic) during skeletal preparation. Archaeozoological materials often does not permit such differentiation [29,39,40].

**Figure 5:**
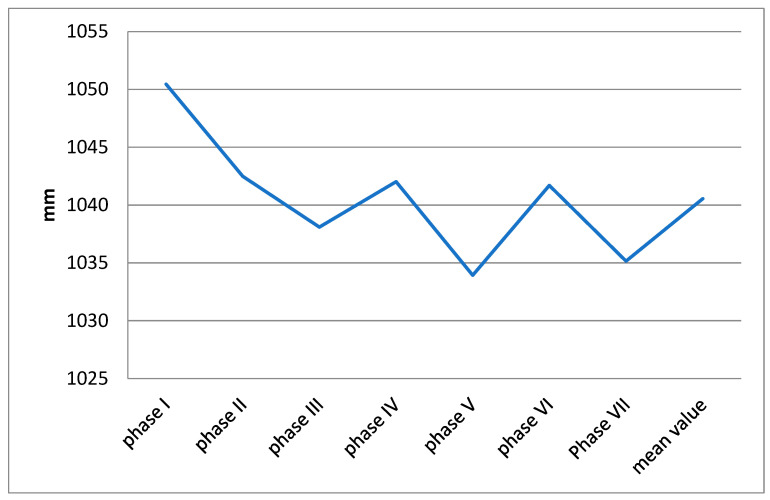
The height at withers (HW-m) estimated on the basis of metapodial osteometry in subsequent phases of chronology [mm] [30].

Because the anatomical position of the bones (within the hand or foot skeleton or the third or fourth digit), could not be determined, the height at withers was calculated using both formulas, and the results were compared (Table 3, Figure 4). Potential methodological error must be acknowledged. Comparison of the resulting HW-p estimates with HW-m calculated using the metapodial method [30] showed that GLpe-based estimates did not yield comparable values in archaeozoological analyses (Figure 5). The use of modern cattle skeletal material to develop the formula of Chrzanowska and Wagner [27] likely accounts for this discrepancy. The range of GLpe values in modern cattle was higher than that in the archaeozoological material. The tested method therefore appears unsuitable for estimating HW-p from smaller proximal phalanges (Table 2). Chrzanowska and Wagner [27] reported a minimum GLpe of 57.1 mm in modern cattle. In the present material, the highest value was 53.28 mm in the seventh phase, indicating that the proximal phalanges were smaller not only in the Middle Ages but also in the early modern period. Lower phenotypic expression of genotype in historical cattle may reflect environmental constraints that prevented husbandry and breeding practices comparable to those of modern intensive production. Numerous studies of medieval and early modern cattle bone remains have reached similar conclusions [1,2,3,4,5,6,7,8,9,10,11,12,13,14,15,16,17,18,19,20,21].

HW-m estimates for cattle bone remains from the same site and historical period were more variable [30]. This variable provided a useful reference and corresponded more closely to changes in domestic animal husbandry in Wrocław (11th–19th c.) than did HW-p. Całkin’s method [31] remained the gold standard in archaeozoology, although it is not without limitations [39]. HW-m values were highest in the first phase of chronology (11th–12th c., beginning of Wrocław’s settlement) (Figure 5). Values were similar in the second, fourth and sixth phase (12th–13th c., 1261 AD–half 14th c. and 14th/15th–17th c., respectively). A marked decrease in HW-m occurred only in the third and fifth phase (13th–1260 AD and half 14th–14th/15th c., respectively). A broader interpretation of these differences was presented in the archaeozoological study of the Wrocław bone assemblage [30]. Here, we emphasize that the principal causes were related to changes in the political and economic status of Wrocław’s burghers over time, indicating the Mongol invasion of 1241 AD and, especially, urban restructuring during the chartering process and the Hussite Wars. These processes were not reflected in HW-p. Without considering HW-m, important evidence concerning human–animal–environment relationships would therefore be lost.

An ideal comparison of available methods for estimating the height at withers would require skeletal remains from the same individuals with known intra-vital height. Because this study analyzed archaeozoological remains representing consumption refuse, these conditions could not be met. The materials accessible for archaeozoological studies are more or less fragmented and smaller phalanges may be better preserved more frequently than larger, complete metapodia. HW-p was calculated using formulas derived from modern bovine skeletal materials from animals of known intra-vital height; however, GLpe values in historical cattle fell below the range values reported by Chrzanowska and Wagner [27].

### 4.3. Proximal Phalanges with Signs of Human Intentional Processing and Use

**Figure 6:**
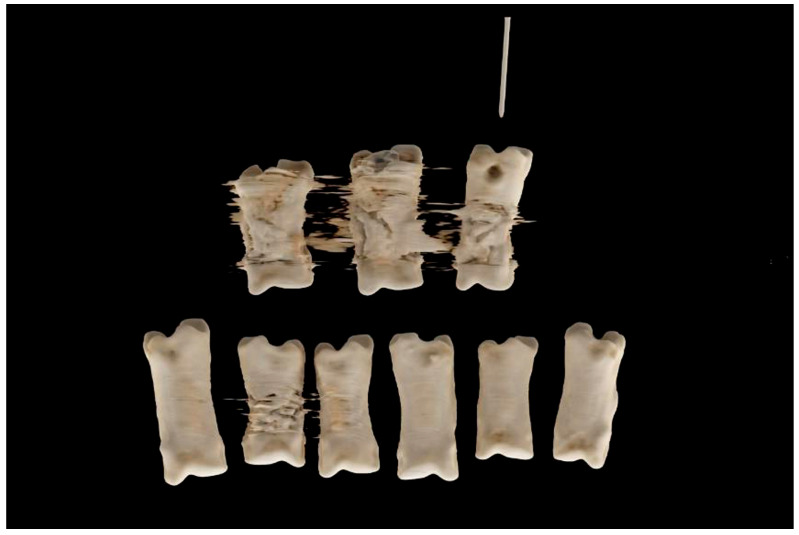
Computed tomography scanning demonstrated metal alloys within the bone medullary cavities, as indicated by imaging artefacts.

**Figure 7:**
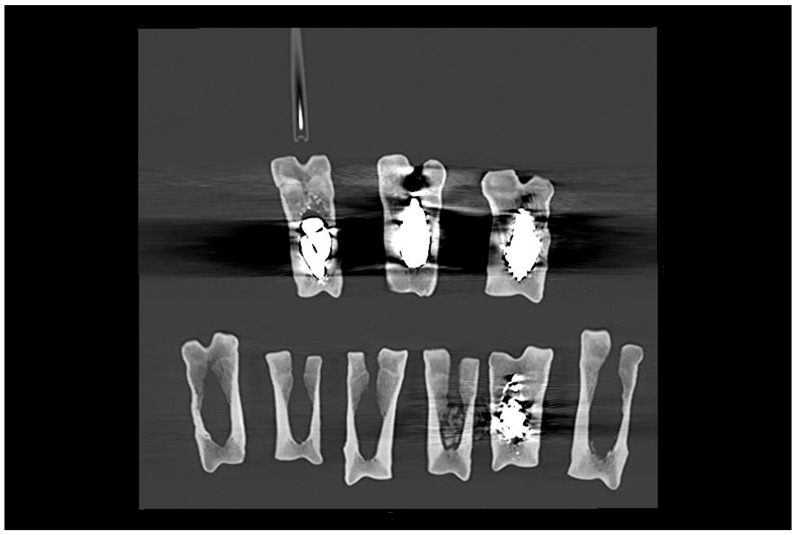
Computed tomography scanning enabled localisation of metal alloys within the bone medullary cavities, as indicated by imaging artefact. In some specimens, the drilled openings are also visible.

The examined bone assemblage included nine phalanges with one or two drilled holes. One possible, although less likely, interpretation is that these modified bones were used as standardized market weights after metal alloy was poured into the holes; however, variation in the masses of the bone specimens argues against standardization. Bläuer et al. [52] proposed another explanation, describing similar artefacts and interpreting them as gaming pieces. The location and archeological context of the findings (including bone and horn workshops, butcher stalls near St. Vitus and St. Catherine streets and New Market square, and continuously occupied burghers plots) do not allow either interpretation to be confirmed [3,4,18,30,48]. The presence of metal alloys within the medullary cavities was identified and confirmed by CT (Figure 6 and Figure 7). The modified phalanges were slender and likely came from young individuals. Table 4 lists the mass of each phalanx, the number of drilled holes, and the presence or absence of an inserted metal weight.

Four artefacts belonged to the fourth phase of chronology, three to the uncertain chronology, and one belonged to the sixth phase. The occurrence of these artefacts in the chartered New Market quarter and burgher plots (1261 AD–half 14th c.), when bone and horn workshops still operated, and in the early modern town (14th/15th–17th c.), after this activity had disappeared, makes a trade-related function less likely and may be more consistent with their interpretation as gaming pieces. Butcher stalls operated in this town quarter during both mentioned historical periods [18,48].

### 4.4. Animal–Human–Environment Relationships

According to Pietruszka and Piekalski [48], domestic animals constituted the most economically important animal group in medieval towns. Similar conclusions emerged from archaeozoological studies of animal bone assemblages from the Wrocław site. These animals supplied a wide range of products, including meat, bone, horn, and hides. The findings indicate that cattle played an important role in both in the proto-urban and urban economies [1,2,3,4,5,30]. However, the available evidence does not establish whether cattle were housed within urban residential plots, in suburban production areas, or in both settings during every phase of urban development. Historical sources also document restrictions on animal husbandry and transportation that were introduced over time [26]. Medieval Wrocław integrated domestic life and economic production within a single spatial framework [53]. Urban plots served not only residential purposes but also accommodated trade, production, and other economic activities. Although available sources do not describe the precise location or architectural form of animal shelters within urban plots, archaeozoological studies show that domestic animals, particularly cattle, were fundamental to the developing urban economy [1,2,3,4,5,48]. By contrast, in rural feudal settlements, the relationship between residential buildings and structures associated with agriculture and livestock can be observed more directly [54]. The morphological examination of bovine proximal phalanges unearthed in the archeological site near St. Vitus and St. Catherine streets and New Market square showed no extensive bone changes characteristic of severe diseases of the locomotor system. Wrocław, the capital city of Silesia, was able to maintain an efficient beef supply that met the burghers’ demand and expectations even during periods of major urban transformation or political instability. This resilience is consistent with the need for both local dukes and rulers of neighbouring kingdoms to consider the city’s political and economic importance. By the modern period, farm animals had largely disappeared from the immediate vicinity of highly urbanized areas. Improved sanitary conditions and the organization of a modern slaughterhouse (1893–1896 AD) marked the beginning of a new era in animal hygiene.

## 5. Conclusions

Morphometric analysis of bovine proximal phalanges demonstrates that these bones can provide valuable information about past human–animal relationships. Their measurements should be interpreted cautiously and in conjunction with findings from other skeletal elements. The analyzed bone remains from the archeological site in Wrocław showed no major morphological changes indicative of heavy labour or severe pathology, suggesting relatively rational cattle use. Hypothetically, livestock trade and imports may have played an important role in supplying meat to the burghers. Additionally, the height at withers estimated from GLpe was not reliable for archaeozoological material because historical cattle differed in size from the modern cattle used to develop the method; therefore, the metapodial method remains the gold standard. Moreover, CT scanning also revealed information beyond conventional assessment of internal bone structure and 3D reconstruction by demonstrating conspicuous imaging interference by metal alloys within the bone.

## Figures and Tables

**Table 1:** Bovine proximal phalanges from the quarter bounded by St. Vitus Street, St. Catherine Street, and New Market square Wrocław.

Phase	Chronology	Interpretation	Number of Artefacts
I	11th–12th c.	Settlement beginnings	10
II	12th–13th c.	*Ad sanctum Adalbertum* craft and market settlement	100
III	13th–1260 AD	*Civitas Vratislavia*, chartered town	38
IV	1261 AD–half 14th c.	Chartered New Market quarter, burgher plots	119
V	half 14th–14th/15th c.	Late medieval city	93
VI	14th/15th–17th c.	Early modern city	19
VII	17th–19th c.	Modern city	20
Uncertain chronology	-	-	38

All measurements were obtained with the electronic slide-calliper to an accuracy of 0.01 cm. Measurement landmarks were defined according to the definition of osteometric points used in archaeozoology [29]. The following dimensions of the bovine proximal phalanges involved: Bp—breadth of the proximal extremity, SB—smallest breadth of the diaphysis, Bd—breadth of the distal extremity, and GLpe—greatest length (peripheral).

**Table 2:** Mean values and standard deviations for the osteometric variables in successive chronological phases [cm]. GLpe—greatest length (peripheral), Bp—breadth of the proximal extremity, SB—smallest breadth of the diaphysis, Bd—breadth of the distal extremity, mean—arithmetic mean value, and SD—standard deviation.

Parameter		Phase I	Phase II	Phase III	Phase IV	Phase V	Phase VI	Phase VII	Uncertain Chronology
GLpe	Mean	49.74	49.71	49.95	49.05	49.59	50.18	53.28	49.46
	SD	1.49	3.10	3.00	2.63	2.76	3.63	4.21	3.74
Bp	Mean	24.47	25.27	24.98	24.93	25.39	25.07	27.10	24.83
	SD	2.97	3.04	2.67	2.52	2.70	3.02	2.89	2.97
SB	Mean	19.74	20.61	20.27	20.44	20.76	20.01	22.25	20.23
	SD	2.56	2.54	2.28	2.17	2.30	2.71	2.41	2.56
Bd	Mean	23.95	23.80	23.52	23.77	23.94	23.04	26.18	23.57
	SD	3.85	2.77	2.61	2.63	2.64	2.73	3.00	2.73

**Table 3:** Height at withers estimated on the basis of proximal phalanx osteometry [cm]. HW-p—height at withers, min—minimal value, max—maximal value, and SD-standard deviation.

Parameter	Phase I	Phase II	Phase III	Phase IV	Phase V	Phase VI	Phase VII	Uncertain Chronology
HW-p	118.04	119.53	119.33	119.33	119.47	119.85	120.89	119.61
Min	93.34	93.34	93.34	93.34	93.34	115.58	117.01	115.76
Max	121.00	123.89	122.36	122.27	122.77	122.57	125.54	124.76
SD	6.12	2.46	3.45	2.30	2.47	1.64	1.62	1.75

**Table 4:** Mass of proximal phalanges, the number of drilled holes, and the presence of an inserted metal weight.

Bone Identification Number	Mass [g]	Number of Holes	Weight: Yes/No
Wita 6/Kat 5 3469/17	15	1	no
Kat 4 4264/17	41	1	yes
NT 9/10 6531/17	27	2	yes
Kat 5 3384/17	13	1	yes
Kat 4 3114/17	19	2	yes
Kat 2 2560/17	21	1	no
NT 11 341/17	17	1	no
Kat 2 3126/17	20	1	no
NT 9/10 1628/17	34	2	yes

## Data Availability

The data presented in this study are available on request from the corresponding author.
